# Diverging roles of the anterior insula in trauma-exposed individuals vulnerable or resilient to posttraumatic stress disorder

**DOI:** 10.1038/s41598-019-51727-3

**Published:** 2019-10-29

**Authors:** Hyeonseok Jeong, Yong-An Chung, Jiyoung Ma, Jungyoon Kim, Gahae Hong, Jin Kyoung Oh, Myeongju Kim, Eunji Ha, Haejin Hong, Sujung Yoon, In Kyoon Lyoo

**Affiliations:** 1 Department of Radiology, Incheon St. Mary’s Hospital, College of Medicine, The Catholic University of Korea, Seoul, South Korea; 2 Ewha Brain Institute, Ewha Womans University, Seoul, South Korea; 3 Department of Brain and Cognitive Sciences, Ewha Womans University, Seoul, South Korea; 4 Graduate School of Pharmaceutical Sciences, Ewha Womans University, Seoul, South Korea; 5 The Brain Institute and Department of Psychiatry, University of Utah, Salt Lake City, Utah USA

**Keywords:** Post-traumatic stress disorder, Risk factors

## Abstract

Distinct brain alterations in response to traumatic events may render trauma-exposed individuals either resilient or vulnerable to posttraumatic stress disorder (PTSD). This study compared regional cerebral metabolic rate of glucose (rCMRglu) among trauma-exposed individuals with current PTSD (PTSD group, *n* = 61), those without current PTSD (Resilience/Recovery group, *n* = 26), and trauma-unexposed controls (Control group, *n* = 54). All participants underwent brain [^18^F]-fluorodeoxyglucose positron emission tomography (FDG-PET) scans. Voxel-wise group differences in rCMRglu among the three groups were evaluated. Associations between rCMRglu and both PTSD severity and resilience were examined. The rCMRglu in the right anterior insula and adjacent prefrontal and striatal areas was lower in the PTSD group, while higher in the Resilience/Recovery group, compared to the Control group. In addition, the lower glucose metabolism of these areas was associated with higher severity and less improvement in PTSD symptoms in the PTSD group, while the higher levels of rCMRglu were correlated with stronger resilience in the Resilience/Recovery group. This study suggests distinct roles of the anterior insula in response to trauma between the PTSD and Resilience/Recovery groups. Heightened rCMRglu in the anterior insular regions may reflect an underlying mechanism of resilience against traumatic stress, while reduced rCMRglu may indicate vulnerability to PTSD.

## Introduction

Exposures to traumatic events are highly prevalent as can be seen from a recent international survey reporting that 70.4% of respondents experienced at least one traumatic event in their lifetime^1^. However, while being exposed to traumatic stress may increase the risk of posttraumatic stress disorder (PTSD)^2^, only a fraction of trauma-exposed individuals culminates in PTSD^3^. This draws attention to the importance of resilience, which can be viewed as a stress coping ability against traumatic stress^4^.

Neuroimaging studies that compare brain structure or function between traumatized participants with and without PTSD may elucidate the neural correlates of both non-adaptive (i.e., pathophysiology of PTSD) and adaptive (i.e., resilience) reactions to trauma exposure. For instance, previous studies have detected significant differences in structural and functional alterations of the brain between trauma-exposed individuals with and without PTSD, as compared with trauma-unexposed controls^5–7^. These results may suggest that the neurobiological characteristics of trauma-exposed individuals show a distinct pattern depending on the development of full-blown PTSD, as opposed to the common belief that neurobiological changes occur along a continuum according to PTSD symptom severity. However, the insufficient number of studies precludes drawing conclusions about the specific brain regions related to the resilience and development of PTSD. Furthermore, despite the potential of [^18^F]-fluorodeoxyglucose positron emission tomography (FDG-PET) in the early detection of brain functional abnormalities prior to apparent structural changes, very few studies using FDG-PET investigated the regional cerebral metabolic rate of glucose (rCMRglu) in trauma-exposed groups, all of which were limited in the number of trauma-exposed subjects^8^.

In this FDG-PET study, we compared rCMRglu at resting condition in a relatively large sample (*n* = 141) composed of trauma-exposed individuals with current PTSD (PTSD group), those without current PTSD (Resilience/Recovery group), and trauma-unexposed controls (Control group). We hypothesized that there would be a different pattern of brain glucose metabolism between trauma-exposed individuals with and without current PTSD. In addition, associations between rCMRglu and clinical characteristics including PTSD symptom severity and resilience were examined in each trauma-exposed group.

## Results

### Demographic and clinical characteristics

Demographic and clinical characteristics of the PTSD (n = 61), Resilience/Recovery (n = 26), and Control groups (n = 54) are shown in Table 1. There were no significant differences in age (*F* = 2.19, *p* = 0.12), sex (*χ*^2^ = 0.07, *p* = 0.96), and handedness (Fisher’s exact test, *p* = 0.40) among the three groups. In the Resilience/Recovery group, 16 (61.5%) participants had been previously diagnosed with PTSD, from which they recovered at the time of the assessment.

**Table 1:** Characteristics of study participants*.

Characteristics	PTSD (*n* = 61)	Resilience/Recovery (*n* = 26)	Control (*n* = 54)	Test statistics
*Demographic characteristics*				
Age, year	42.1 ± 11.6	47.3 ± 11.9	44.7 ± 9.8	*F* = 2.19, *p* = 0.12
Women	43 (70.5)	19 (73.1)	38 (70.4)	*χ*^2^ = 0.07, *p* = 0.96
Right handedness	57 (93.4)	24 (92.3)	53 (98.1)	*p* = 0.40
*Clinical characteristics*				
Time since the index trauma, month	6.2 ± 4.4	8.2 ± 4.9	N/A	*t* = 1.93, *p* = 0.06
Type of index trauma				*p* = 0.47
Physical violence	15 (24.6)	8 (30.8)	N/A	
Sexual violence	11 (18.0)	3 (11.5)	N/A	
Life-threatening accident/injury	4 (6.6)	4 (15.4)	N/A	
Others	31 (50.8)	11 (42.3)	N/A	
Frequency of trauma exposure				*p* = 1.00
Single exposure	51 (83.6)	22 (84.6)	N/A	
Repeated exposure	10 (16.4)	4 (15.4)	N/A	
Type of trauma exposure				*χ*^2^ = 0.58, *p* = 0.45
Direct exposure	42 (68.9)	20 (76.9)	N/A	
Indirect exposure	19 (31.2)	6 (23.1)	N/A	
Type of sexual violence				*p* = 0.19
Rape	4 (36.4)	3 (100.0)	N/A	
Sexual assault other than rape	7 (63.6)	0 (0.0)	N/A	
Psychotropic medication use^†^				
Antidepressants	14 (23.0)	0 (0.0)	0 (0.0)	
Antipsychotics/mood stabilizer	0 (0.0)	0 (0.0)	0 (0.0)	
Anxiolytics	16 (26.2)	0 (0.0)	0 (0.0)	
Others	2 (3.3)	0 (0.0)	0 (0.0)	

*Data are presented as mean ± standard deviation or number (%).

^†^Participants taking more than one type of psychotropic medications were counted in each category.

Control, trauma-unexposed individuals; N/A, not applicable; PTSD, posttraumatic stress disorder.

There were no significant differences in clinical characteristics including time since the index trauma (*t* = 1.93, *p* = 0.06), type of index trauma (Fisher’s exact test, *p* = 0.47), frequency of trauma exposure (single vs. repeated, Fisher’s exact test, *p* = 1.00), type of trauma exposure (direct vs. indirect, *χ*^2^ = 0.58, *p* = 0.45), and type of sexual violence (rape vs. sexual assault other than rape, Fisher’s exact test, *p* = 0.19) between the PTSD and Resilience/Recovery groups. In addition, 17 (27.9%) participants in the PTSD group reported concurrent use of psychotropic medication at the time of the assessment, while none from the Resilience/Recovery and Control groups were under concurrent use of psychotropic medication.

**Figure 1:**
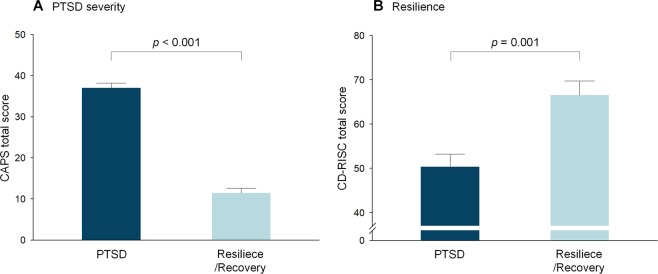
Comparisons of (**A**) PTSD symptom severity and (**B**) resilience levels between the PTSD and Resilience/Recovery groups. Error bars indicate standard errors. CAPS, Clinician-Administered PTSD Scale; CD-RISC, Connor‐Davidson Resilience Scale; PTSD, posttraumatic stress disorder.

The PTSD group showed higher total scores of the Clinician-Administered PTSD Scale (CAPS) for Diagnostic and Statistical Manual of Mental Disorders (DSM)-5 (*t* = 13.3, *p* < 0.001), and lower total scores of the Connor-Davidson Resilience Scale (CD-RISC) (n = 53 vs. 24, *t* = −3.42, *p* = 0.001), compared to the Resilience/Recovery group (Fig. 1).

### Group differences in rCMRglu

**Figure 2:**
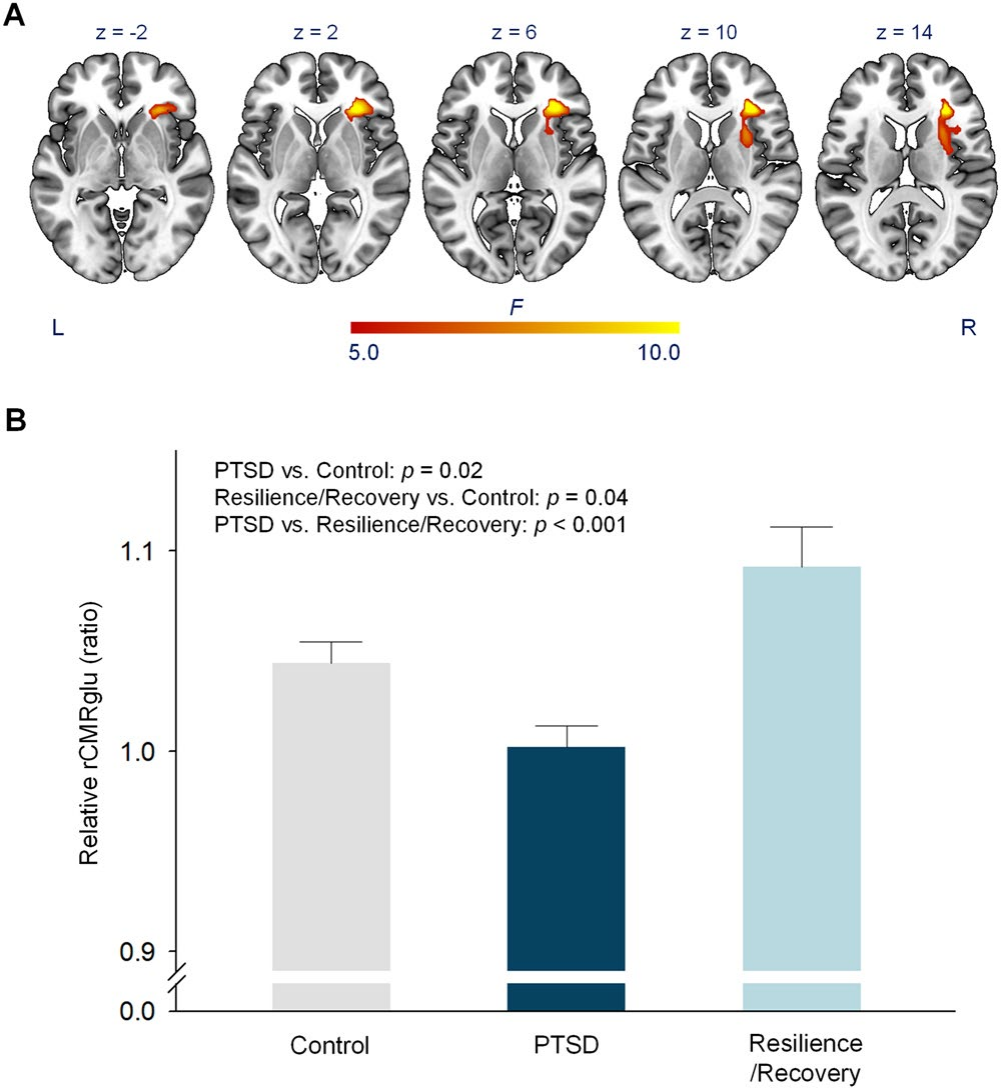
Group differences of rCMRglu. (**A**) Brain regions with significant differences in relative rCMRglu across the three groups (PTSD vs. Resilience/Recovery vs. Control groups). An analysis of covariance F-test was conducted. The primary threshold was *p* < 0.005 and the extent threshold was family-wise error corrected *p* < 0.05. The color bar represents voxel-level *F* values. The numbers above brain slices indicate z coordinates in the Montreal Neurological Institute space. (**B**) The bar graph for the relative rCMRglu in the significant cluster among the three groups. Error bars indicate standard errors and *p* values were derived from post-hoc pairwise comparisons using Scheffe’s test. Control, trauma-unexposed individuals; L, left; PTSD, posttraumatic stress disorder; rCMRglu, regional cerebral metabolic rate of glucose; R, right.

In the voxel-wise analysis of covariance (ANCOVA) analysis among the three groups, we found a significant cluster in the right anterior insula and its adjacent areas including the frontal operculum, inferior frontal and orbitofrontal cortices, and putamen (*F* = 14.57, *p* < 0.001, cluster size = 733 voxels, peak coordinates = 30, 30, 12) (Fig. 2A). Post-hoc analysis for pairwise group comparisons demonstrated that the mean rCMRglu of the significant cluster was lower in the PTSD group as compared with the Control group (*p* = 0.02), whereas the mean rCMRglu of the same cluster was higher in the Resilience/Recovery group than in the Control group (*p* = 0.04). As expected, there was a significant difference in the mean rCMRglu of the abovementioned cluster between the PTSD and Resilience/Recovery groups (*p* < 0.001) (Fig. 2B).

The results from the exploratory pairwise comparisons of rCMRglu are presented in the Supplementary Information. Compared to the Control group, the Resilience/Recovery group demonstrated increased rCMRglu in the inferior frontal, orbitofrontal, insular, frontal medial, and lateral occipital cortices (Supplementary Fig. 1 and Supplementary Table 1), whereas the PTSD group showed decreased rCMRglu in the orbitofrontal, insular, temporal fusiform, parahippocampal, parietal opercular, and supramarginal cortices (Supplementary Fig. 2 and Supplementary Table 2). Compared to the PTSD group, the Resilience/Recovery group revealed higher rCMRglu in the inferior frontal, insular, angular, temporal/occipital fusiform, lateral occipital, superior parietal, inferior/middle temporal, and frontal pole areas (Supplementary Fig. 3 and Supplementary Table 3).

### Relationships between rCMRglu and clinical characteristics

**Figure 3:**
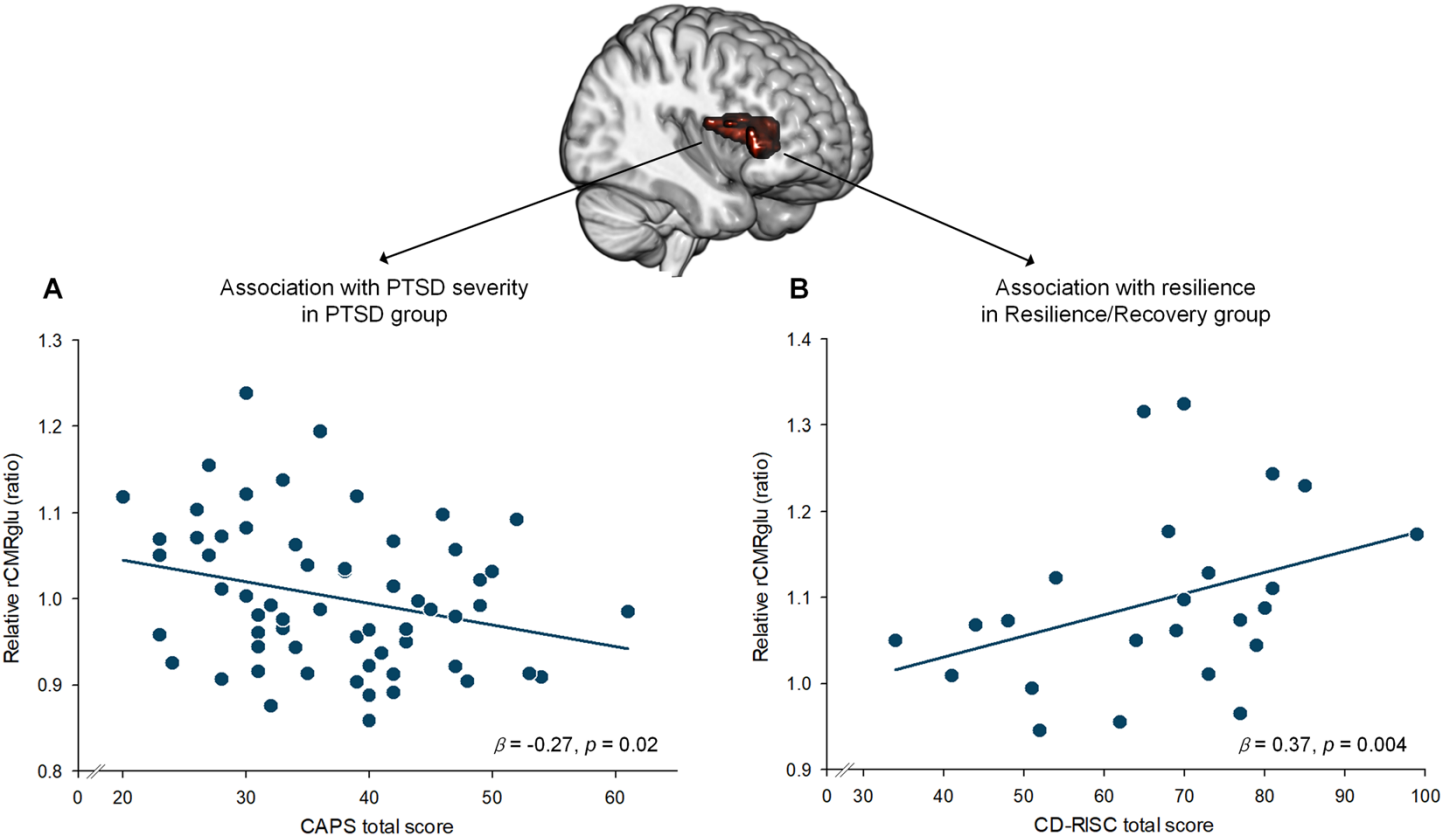
Associations between the rCMRglu in the right anterior insula cluster and (**A**) the CAPS total scores in the PTSD group and (**B**) the CD-RISC total scores in the Resilience/Recovery group. There was a significant relationship between the CAPS total scores and the rCMRglu in the right anterior insula in the PTSD group (*β* = −0.27, *p* = 0.02). However, this relationship was not observed in the Resilience/Recovery group (*β* = 0.16, *p* = 0.45, Supplementary Fig. 4). A positive relationship between the CD-RISC total scores and the rCMRglu in the right anterior insula was observed in the Resilience/Recovery group (*β* = 0.37, *p* = 0.004), but not in the PTSD group (*β* = 0.18, *p* = 0.23, Supplementary Fig. 6). The solid lines indicate regression lines. CAPS, Clinician-Administered PTSD Scale; CD-RISC, Connor‐Davidson Resilience Scale; PTSD, posttraumatic stress disorder; rCMRglu, regional cerebral metabolic rate of glucose.

For the association between rCMRglu and PTSD symptom severity, a significant negative correlation between the rCMRglu in the anterior insula cluster and the CAPS total scores was found in the PTSD group (*β* = −0.27, *p* = 0.02) (Fig. 3A), but not in the Resilience/Recovery group (*β* = 0.16, *p* = 0.45) (Supplementary Fig. 4). In addition, the rCMRglu in the same cluster showed a significant negative correlation with changes in the CAPS total scores in the PTSD group (*β* = −0.29, *p* = 0.01), while a significant correlation was not found in the Resilience/Recovery group (*β* = 0.05, *p* = 0.80) (Supplementary Fig. 5).

For the relationship between rCMRglu and resilience, a positive correlation was found between the rCMRglu in the anterior insular cluster and the total scores of the CD-RISC in the Resilience/Recovery group (*β* = 0.37, *p* = 0.004) (Fig. 3B), but not in the PTSD group (*β* = 0.18, *p* = 0.23) (Supplementary Fig. 6).

The results from the abovementioned correlational analyses remained unchanged even with time since the index trauma, type of index trauma, and concurrent use of psychotropic medication included as an additional covariate, respectively (Supplementary Table 4).

## Discussion

To our knowledge, this is the largest FDG-PET study to date that investigated differences in rCMRglu among trauma-exposed individuals according to the presence of PTSD. The voxel-wise analysis revealed that rCMRglu in the right anterior insula and its adjacent areas was lower in the PTSD group, while higher in the Resilience/Recovery group, compared to the Control group. Notably, the lower glucose metabolism of these areas was associated with both higher severity and less improvement in PTSD symptoms in the PTSD group, while the higher levels of rCMRglu were correlated with stronger resilience in the Resilience/Recovery group. Exploratory pairwise comparisons demonstrated higher glucose metabolism in the prefrontal regions of the Resilience/Recovery group and lower rCMRglu in the temporal and parietal cortices of the PTSD group as compared to the Control group.

The current finding of PTSD-related rCMRglu reduction in the anterior insula and prefrontal regions provides supportive evidence for the significant role of the insula towards posttraumatic stress, and is in line with a previous FDG-PET study on PTSD patients which showed decreased basal glucose metabolism in the insula and prefrontal cortex^9^. The reduction in rCMRglu among these regions may also support a previous functional magnetic resonance imaging (MRI) study on PTSD patients that reported reduced baseline amplitude of low-frequency fluctuation (ALFF), which reflects intrinsic functional activity of the brain^10^. It is also noteworthy that the current result on insular activity at resting-state contrasts with previous findings under task conditions, where a meta-analysis of functional neuroimaging studies in PTSD patients indicated hyperactivity of the insula during negative emotional processing^11^. Furthermore, previous research reported that in traumatic conditions as compared to neutral conditions, regional cerebral blood flow (rCBF) in the orbitofrontal and insular cortices is increased in PTSD patients^12^. Together, it can be suggested that the direction of alteration in insular activity is distinct based on whether the brain is under a resting-state or task-based condition, and whether the task at hand is negative in nature.

The anterior insula plays a pivotal role as an interface between interoceptive awareness of body states and subjective emotional experiences^13^. In addition, this region is closely related to anticipation of aversive stimuli, threat detection, and fear generalization^14^. Functional abnormalities in this brain region such as heightened activity to salient stimuli may trigger exaggerated prediction signal of an aversive body state and subsequent anxious mood, worrisome thoughts, and other avoidance behaviors^15^. As a result, this process may increase the risk of anxiety disorders^15^. For instance, PTSD patients showed increased activation of the anterior insula during anticipation of aversive stimuli and during processing of threat-related emotion compared to non-traumatized controls^16,17^. Prominent increased activation of the anterior insula may also underlie somatic symptoms and heightened attention to interoceptive states, both of which are frequently observed in PTSD patients^18^. Taken together, our results suggest that PTSD patients may have lower baseline activity of the anterior insula, while in contrast have higher activation levels during emotional processing of negative or traumatic stimuli.

On the other hand, the trauma-exposed individuals without current PTSD demonstrated increased baseline rCMRglu in the right anterior insula and surrounding areas compared to the trauma-unexposed controls. Furthermore, the glucose metabolism was positively associated with resilience. A previous functional MRI study showed higher ALFF of the prefrontal cortex, insula, and putamen in trauma-exposed individuals without PTSD^19^. In healthy volunteers, stronger resting-state functional connectivity of the anterior insula within the salience network was associated with more resilient personality^20^. Interestingly, high-resilient and low-resilient individuals may exhibit differential activation patterns of the anterior insula to aversive or neutral pictures after threat cues. Specifically, high-resilient participants showed activation only to aversive pictures which was followed by quickly returning to baseline, whereas low-resilient volunteers demonstrated more prolonged activation to both aversive and neutral pictures^21^. In addition to the potential roles of appropriate insular activation patterns against negative emotional processing in resilience, the current findings suggest an association between stronger resilience and higher baseline activity of the anterior insula.

Brain areas with significant group differences also included the inferior frontal and orbitofrontal cortices and putamen, all of which have been known for their involvement in fear extinction, emotional regulation, and pathophysiology of PTSD^22–24^. For instance, task-evoked activation of the inferior frontal gyrus was negatively associated with symptoms of reexperience, avoidance, and dissociation in response to script-driven trauma imagery^23^. During response inhibition, PTSD patients showed a reduced response in the inferior frontal gyrus to contextual cues^25^. In addition, emotional numbing, a commonly observed symptom in PTSD patients, was correlated with striatal hypoactivation to happy facial expressions^22^. On the other hand, levels of activation in the orbitofrontal cortex were found to be positively correlated with resilience during trauma-related imagery paradigm in trauma-unexposed individuals^26^.

In addition to the anterior insula and adjacent areas, additional analysis revealed higher rCMRglu in the prefrontal regions of the Resilience/Recovery group and lower glucose metabolism in the parahippocampal, temporal fusiform, and supramarginal gyrus of the PTSD group, compared with the Control group. This finding is in alignment with a previous study which found reduced rCBF in the parahippocampal gyrus and inferior parietal lobule of PTSD patients during imagery of traumatic events^12^. In addition, hypoactivity of the ventral stream of the visual system was also reported in patients with PTSD during a picture-viewing task^27^. Therefore, hypometabolism of the parahippocampal gyrus may be associated with altered processing of autobiographical memories^28^ and metabolic deficits of the fusiform and supramarginal gyrus may be involved in abnormal visual and sensory processing in PTSD patients. In fact, PTSD patients often feel overwhelmed or insecure in the face of complex sensory inputs due to disrupted sensory filtering^29^.

Limitations of the current study should be addressed. First, the current study aimed to explore the neural correlates of resilience in the case of trauma through the Resilience/Recovery group, which included the collective recruitment of trauma-exposed individuals both with and without lifetime PTSD. This criterion allows to investigate both the resilient nature which protects against PTSD pathophysiology, as well as persistent resilience that allows the recovery from PTSD. However, it is noteworthy that these two populations may have distinct clinical and neurobiological characteristics^30^. Therefore, future studies that consider these two subgroups separately may further elucidate the detailed neurobiological pathways of resilience towards trauma. Second, although there were no differences in the characteristics of the index trauma between the PTSD and Resilience/Recovery groups, an objective measure for evaluating the severity of the trauma would be necessary to examine its potential synergic or interactive influences on PTSD-related alterations in glucose metabolism. Third, although the sensitivity analyses showed that concurrent use of psychotropic medication did not significantly change the correlations between rCMRglu and clinical characteristics in the PTSD group, the group difference in medication status may have influenced our results. Lastly, due to the cross-sectional nature of the current study design, it cannot be determined whether the diverging patterns of rCMRglu in the two trauma-exposed groups were pre-existing or acquired characteristics.

In conclusion, our findings suggest that the anterior insula may play a distinct role in the physiological response to traumatic stress. The heightened glucose metabolism in this area may be an indicator of a possible mechanism for resilience against traumatic stress in trauma-exposed individuals who are more resilient, while the reduced glucose metabolism may underlie vulnerability to PTSD development in trauma-exposed individuals. Further longitudinal studies are warranted to investigate whether rCMRglu in the anterior insula can be useful for prediction of clinical progression in these populations.

## Methods

### Participants

A total of 141 subjects aged between 20 and 65 years were recruited and classified into three groups based on clinical assessments including history taking of trauma and current diagnosis of PTSD: 61 trauma-exposed individuals with current PTSD (PTSD group), 26 trauma-exposed individuals without current PTSD (Resilience/Recovery group), and 54 non-traumatized controls (Control group). The trauma-exposed individuals (*n* = 87) were enrolled in the study 6.8 ± 4.6 months after any direct or indirect exposures to trauma, such as actual or threatened death, serious injury, or sexual violence. The exclusion criteria were as follows: 1) significant medical conditions; 2) lifetime Axis 1 psychiatric disorders including PTSD (prior to the index trauma for trauma-exposed groups), psychotic disorders, or bipolar disorder; 3) Axis 2 antisocial or borderline personality disorders; 4) a history of traumatic brain injury with loss of consciousness; and 5) contraindications to brain MRI and PET including metal implants and pregnancy. All participants provided written informed consent prior to enrollment in the study. This study was approved by the Institutional Review Board of Ewha Womans University. All experimental procedures and methods were carried out in accordance with institutional and national guidelines and regulations.

### Clinical assessment

The screening for any mental disorders was conducted using the Structured Clinical Interview (SCID) for DSM-IV^31^. The CAPS for DSM-5 was used for the diagnosis of PTSD as well as evaluation of PTSD symptom severity^32^. The CD-RISC was used to evaluate resilience, in which higher total scores reflect greater resilience^33^.

### Image acquisition

Brain FDG-PET scans were performed using a Discovery STE PET-CT scanner (GE Healthcare, Milwaukee, WI, USA). All participants fasted for 6 hours to ensure that their blood glucose level was < 120 mg/dl and were intravenously injected with 185—259 MBq of FDG. Participants were imaged after 40 minutes of uptake period during which they were awake and rested in supine position in a dark and quiet room with their eyes closed. After acquiring the computed tomography (CT) scans for attenuation correction, PET images were acquired in 128 × 128 matrices with a reconstructed voxel size of 1.95 × 1.95 × 3.27 mm. Image reconstruction was conducted using an ordered subset expectation maximization algorithm^34^. The total scan time was 15 minutes.

High-resolution T1-weighted MPRAGE as well as fluid-attenuated inversion recovery (FLAIR) images were acquired using a 3.0 Tesla MR scanner (Philips Medical Systems, Best, The Netherlands) for the purpose of image registration and screening for gross brain abnormalities, respectively, with the following parameters: sagittal T1-weighted images, echo time (TE) = 3.4 ms, repetition time (TR) = 7.4 ms, flip angle (FA) = 8°, field of view (FOV) = 22 × 22 cm, matrix = 256 × 256, voxel size = 1.00 × 0.86 × 0.86 mm; FLAIR images, TE = 278 ms, TR = 4,800 ms, inversion time = 1,650 ms, FOV = 24 × 24 cm, matrix = 240 × 240, voxel size = 1.0 × 1.0 × 1.0 mm.

### Image processing

Statistical Parametric Mapping (SPM) 12 (Wellcome Department of Cognitive Neurology, Institute of Neurology, London, UK; http://www.fil.ion.ucl.ac.uk/spm) was used for preprocessing of the image data. Each FDG-PET image was coregistered to the corresponding T1-weighted image. After each T1-weighted image was spatially normalized to the standard SPM T1 template (Montreal Neurological Institute, McGill University, Montreal, Canada), the resulting transformation parameters were applied to the coregistered PET data. All PET scans were then resliced with a voxel size of 2.0 × 2.0 × 2.0 mm and smoothed with an 8 mm full width at half-maximum (FWHM) isotropic Gaussian kernel. Voxel intensity at each voxel was scaled to the mean cerebellar uptake using proportional scaling in order to obtain relative rCMRglu as a ratio, since cerebellar glucose metabolism is reported to be least affected by aging^35^.

Voxel-wise differences in rCMRglu among the three groups were examined using ANCOVA F-test with age and sex as covariates. The primary threshold was set at *p* < 0.005 and the extent threshold was set at a family-wise error (FWE) corrected *p* < 0.05. Mean rCMRglu values were extracted from significant clusters using the MarsBar toolbox (http://marsbar.sourceforge.net)^36^ and post-hoc pairwise comparisons were carried out with Scheffe’s test.

For exploratory purposes, voxel-wise analyses were performed to compare rCMRglu between the PTSD vs. Control, the Resilience/Recovery vs. Control, and the PTSD vs. Resilience/Recovery groups, respectively, using separate ANCOVA tests with age and sex as covariates. The primary threshold was *p* < 0.005 and extent threshold was 100 or more contiguous voxels.

### Statistical analysis

For demographic and clinical measures, continuous variables were compared using independent t-test or one-way analysis of variance (ANOVA). Differences in categorical variables were assessed using chi-square test or Fisher’s exact test.

For each trauma-exposed group, linear regression was performed between the mean rCMRglu extracted from the significant cluster and clinical variables, including the CAPS and CD-RISC total scores. In addition, associations between the abovementioned rCMRglu and changes in the CAPS total scores (current scores - lifetime scores) were examined using linear regression in each trauma-exposed group. To evaluate potential confounding effects, all correlation analyses were repeated using time since the index trauma, type of index trauma, and concurrent use of psychotropic medication as an additional covariate, respectively.

A two-tailed *p* < 0.05 was considered statistically significant. All statistical analyses were conducted using Stata version 13.1 (StataCorp., College Station, TX, USA). All datasets generated and/or analyzed during the current study are available from the corresponding author on reasonable request.

## Supplementary information

Supplementary Information
