# Supplementary material for: Diverging roles of the anterior insula in trauma-exposed individuals vulnerable or resilient to posttraumatic stress disorder
Source: Sci Rep. 2019 Oct 29;9:15539. doi: 10.1038/s41598-019-51727-3 (PMC6820768; doi:10.1038/s41598-019-51727-3)
Supplement: Supplementary file 1 — Supplementary Information [file 41598_2019_51727_MOESM1_ESM.docx]

***Supplementary Information for***

**Diverging roles of the anterior insula in trauma-exposed individuals**

**vulnerable or resilient to posttraumatic stress disorder**

Hyeonseok Jeong^*^, Yong-An Chung^*^, Jiyoung Ma, Jungyoon Kim, Gahae Hong, Jin Kyoung Oh, Myeongju Kim, Eunji Ha, Haejin Hong, Sujung Yoon^†^, In Kyoon Lyoo^†^

^*^ These authors contributed equally to this work

^†^ To whom correspondence should be addressed

In Kyoon Lyoo, MD, PhD, MMS

Ewha Brain Institute and Department of Brain and Cognitive Sciences, Ewha Womans University

52 Ewhayeodae-gil, Seodaemun-gu, Seoul, 03760, South Korea

Tel: +82-2-3277-6550, Fax: +82-2-3277-6562, E-mail: inkylyoo@ewha.ac.kr

Sujung Yoon, MD, PhD

Ewha Brain Institute and Department of Brain and Cognitive Sciences, Ewha Womans University

52 Ewhayeodae-gil, Seodaemun-gu, Seoul, 03760, South Korea

Tel: +82-2-3277-6564, Fax: +82-2-3277-6562, E-mail: sujungjyoon@ewha.ac.kr

**Supplementary Table 1.** Brain regions showing significant differences in regional cerebral metabolic rate of glucose between trauma-exposed individuals without current PTSD (Resilience/Recovery group) and trauma-unexposed controls (Control group)

| Brain region | Side | Peak coordinates | | | Cluster size (voxels) | Voxel-level | |
| --- | --- | --- | --- | --- | --- | --- | --- |
|  |  | x | y | z |  | Peak  *t* value | Peak  *p* value |
| *Resilience/Recovery > Control* | | | | | | | |
| Inferior frontal gyrus | L | -34 | 20 | 16 | 178 | 4.04 | < 0.001 |
| Frontal pole,  frontal orbital cortex,  insular cortex | R | 28 | 32 | 12 | 524 | 3.71 | < 0.001 |
| Frontal medial cortex | L | -12 | 50 | -12 | 228 | 3.64 | < 0.001 |
| Lateral occipital cortex | L | -34 | -64 | 16 | 185 | 3.50 | < 0.001 |
|  |  |  |  |  |  |  |  |
| *Resilience/Recovery < Control* | | | | | | | |
| None |  |  |  |  |  |  |  |

An analysis of covariance was conducted with age and sex as covariates. The primary threshold was *p* < 0.005 and extent threshold was set at 100 or more contiguous voxels. Significant brain regions were identified using the Harvard-Oxford atlas.

L, left; PTSD, posttraumatic stress disorder; R, right.

**Supplementary Table 2.** Brain regions showing significant differences in regional cerebral metabolic rate of glucose between trauma-exposed individuals with current PTSD (PTSD group) and trauma-unexposed controls (Control group)

| Brain region | Side | Peak coordinates | | | Cluster size (voxels) | Voxel-level | |
| --- | --- | --- | --- | --- | --- | --- | --- |
|  |  | x | y | z |  | Peak  *t* value | Peak  *p* value |
| *PTSD* > *Control* | | | | | | | |
| None |  |  |  |  |  |  |  |
|  |  |  |  |  |  |  |  |
| *PTSD* < *Control* | | | | | | | |
| Frontal orbital cortex,  insular cortex | R | 30 | 28 | 0 | 216 | 3.88 | < 0.001 |
| Temporal fusiform cortex, parahippocampal gyrus | L | -38 | -34 | -18 | 174 | 3.81 | < 0.001 |
| Temporal fusiform cortex,  parahippocampal gyrus | L | -38 | -12 | -30 | 105 | 3.57 | < 0.001 |
| Temporal fusiform cortex,  parahippocampal gyrus | R | 40 | -12 | -28 | 150 | 3.40 | < 0.001 |
| Parietal operculum cortex,  supramarginal gyrus | R | 48 | -22 | 20 | 278 | 3.28 | 0.001 |

An analysis of covariance was conducted with age and sex as covariates. The primary threshold was *p* < 0.005 and extent threshold was set at 100 or more contiguous voxels. Significant brain regions were identified using the Harvard-Oxford atlas.

L, left; PTSD, posttraumatic stress disorder; R, right.

**Supplementary Table 3.** Brain regions showing significant differences in regional cerebral metabolic rate of glucose between trauma-exposed individuals with current PTSD (PTSD group) and those without current PTSD (Resilience/Recovery group)

| Brain region | Side | Peak coordinates | | | Cluster size (voxels) | Voxel-level | |
| --- | --- | --- | --- | --- | --- | --- | --- |
|  |  | x | y | z |  | Peak  *t* value | Peak  *p* value |
| *Resilience/Recovery > PTSD* | | | | | | | |
| Inferior frontal gyrus,  insular cortex,  frontal opercular cortex | R | 30 | 30 | 12 | 1,460 | 5.30 | < 0.001 |
| Inferior frontal gyrus,  central opercular cortex,  frontal orbital cortex | L | -36 | 22 | 14 | 975 | 4.84 | < 0.001 |
| Angular gyrus | R | 44 | -54 | 18 | 412 | 3.92 | < 0.001 |
| Temporal fusiform cortex, | L | -36 | -30 | -14 | 564 | 3.75 | < 0.001 |
| Lateral occipital cortex,  middle temporal gyrus | L | -36 | -72 | 18 | 248 | 3.73 | < 0.001 |
| Superior parietal lobule,  pre/postcentral gyrus | L | -22 | -44 | 58 | 259 | 3.71 | < 0.001 |
| Inferior/middle temporal gyrus | R | 46 | -36 | -22 | 275 | 3.70 | < 0.001 |
| Angular gyrus | L | -40 | -54 | 32 | 201 | 3.65 | < 0.001 |
| Occipital fusiform gyrus | R | 30 | -64 | -10 | 111 | 3.52 | < 0.001 |
| Frontal pole | R | 22 | 46 | 28 | 111 | 3.46 | < 0.001 |
| Frontal pole | R | 32 | 48 | -6 | 148 | 3.31 | 0.001 |
| Temporal fusiform cortex | L | -38 | -12 | -32 | 105 | 3.12 | 0.001 |
|  |  |  |  |  |  |  |  |
| *Resilience/Recovery < PTSD* | | | | | | | |
| None |  |  |  |  |  |  |  |

An analysis of covariance was conducted with age and sex as covariates. The primary threshold was *p* < 0.005 and extent threshold was set at 100 or more contiguous voxels. Significant brain regions were identified using the Harvard-Oxford atlas.

L, left; PTSD, posttraumatic stress disorder; R, right.

**Supplementary Table 4.** Correlations between clinical characteristics and regional cerebral metabolic rate of glucose in the right anterior insula cluster

| Independent variable | PTSD group | Resilience/Recovery group |  |
| --- | --- | --- | --- |
| *Covariate: none* |  |  | |
| CAPS total score | *β* = -0.27, *p* = 0.02 | *β* = 0.16, *p* = 0.45 | |
| Changes in CAPS total score | *β* = -0.29, *p* = 0.01 | *β* = 0.05, *p* = 0.80 | |
| CD-RISC total score | *β* = 0.18, *p* = 0.23 | *β* = 0.37, *p* = 0.004 | |
|  |  |  | |
| *Covariate: time since the index trauma* |  |  | |
| CAPS total score | *β* = -0.27, *p* = 0.02 | *β* = 0.23, *p* = 0.30 | |
| Changes in CAPS total score | *β* = -0.33, *p* = 0.01 | *β* = -0.01, *p* = 0.97 | |
| CD-RISC total score | *β* = 0.17, *p* = 0.27 | *β* = 0.31, *p* = 0.03 | |
|  |  |  | |
| *Covariate: types of index trauma* |  |  | |
| CAPS total score | *β* = -0.26, *p* = 0.02 | *β* = 0.15, *p* = 0.55 | |
| Changes in CAPS total score | *β* = -0.30, *p* = 0.01 | *β* = 0.03, *p* = 0.87 | |
| CD-RISC total score | *β* = 0.13, *p* = 0.38 | *β* = 0.38, *p* = 0.01 | |
|  |  |  | |
| *Covariate: concurrent use of psychotropic medication* | | | |
| CAPS total score | *β* = -0.25, *p* = 0.04 | N/A | |
| Changes in CAPS total score | *β* = -0.28, *p* = 0.02 | N/A | |
| CD-RISC total score | *β* = 0.13, *p* = 0.41 | N/A | |

CAPS, Clinician-Administered PTSD Scale; CD-RISC, Connor‐Davidson Resilience Scale; N/A, not applicable; PTSD, posttraumatic stress disorder.


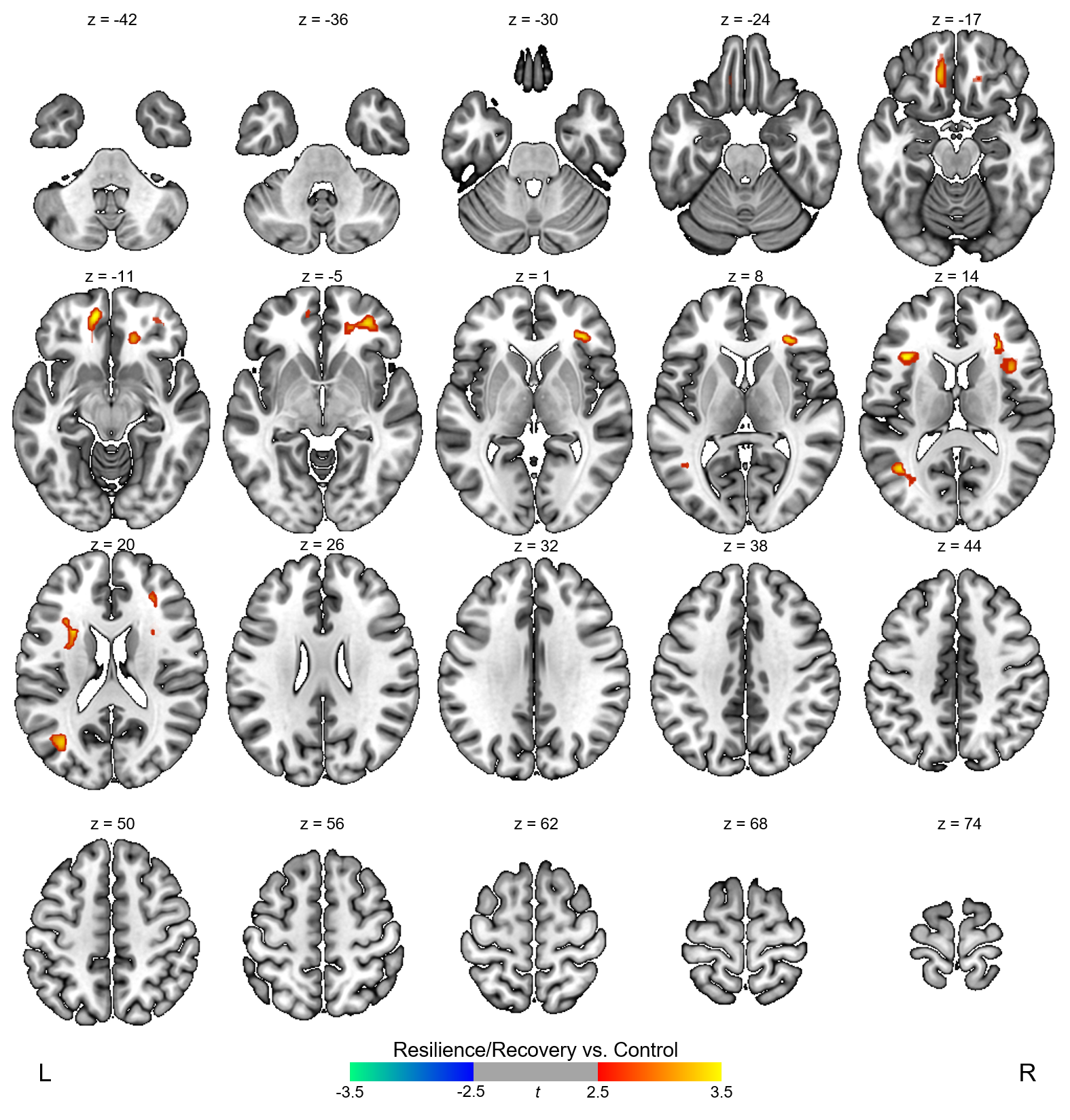


**Supplementary Figure 1.** Brain regions with higher (red-yellow) or lower (blue-green) regional cerebral metabolic rate of glucose in trauma-exposed individuals without current PTSD (Resilience/Recovery group) in comparison with trauma-unexposed controls (Control group). An analysis of covariance was conducted with age and sex as covariates. The primary threshold was *p* < 0.005 and extent threshold was set at 100 or more contiguous voxels. The color bar represents voxel-level *t* values. The numbers above the brain slices indicate z coordinates in the Montreal Neurological Institute space. Detailed cluster information is listed in the Supplementary Table 1.

L, left; PTSD, posttraumatic stress disorder; R, right.


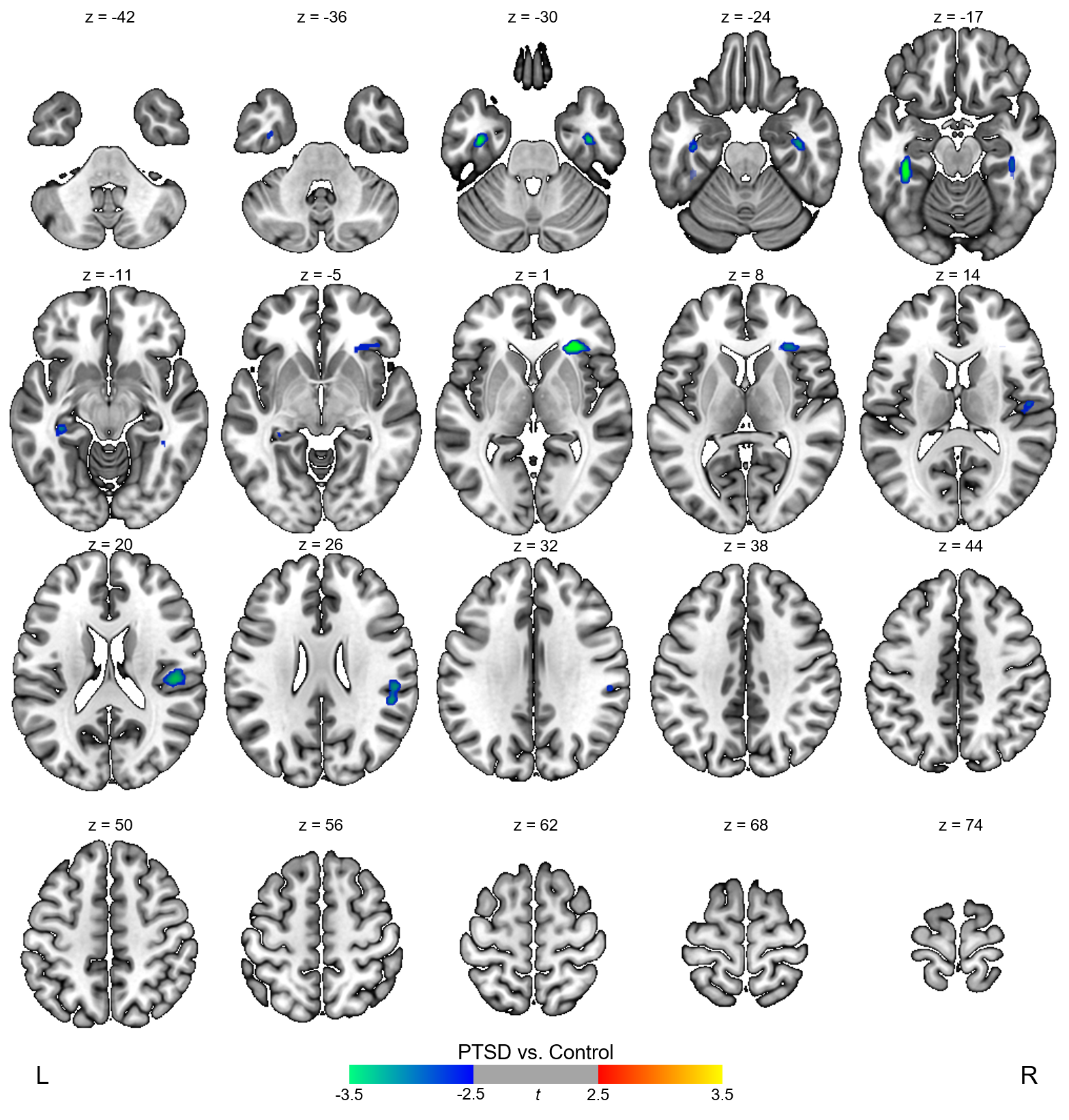


**Supplementary Figure 2.** Brain regions with higher (red-yellow) or lower (blue-green) regional cerebral metabolic rate of glucose in trauma-exposed individuals with current PTSD (PTSD group) in comparison with trauma-unexposed controls (Control group). An analysis of covariance was conducted with age and sex as covariates. The primary threshold was *p* < 0.005 and extent threshold was set at 100 or more contiguous voxels. The color bar represents voxel-level *t* values. The numbers above the brain slices indicate z coordinates in the Montreal Neurological Institute space. Detailed cluster information is listed in the Supplementary Table 2.

L, left; PTSD, posttraumatic stress disorder; R, right.


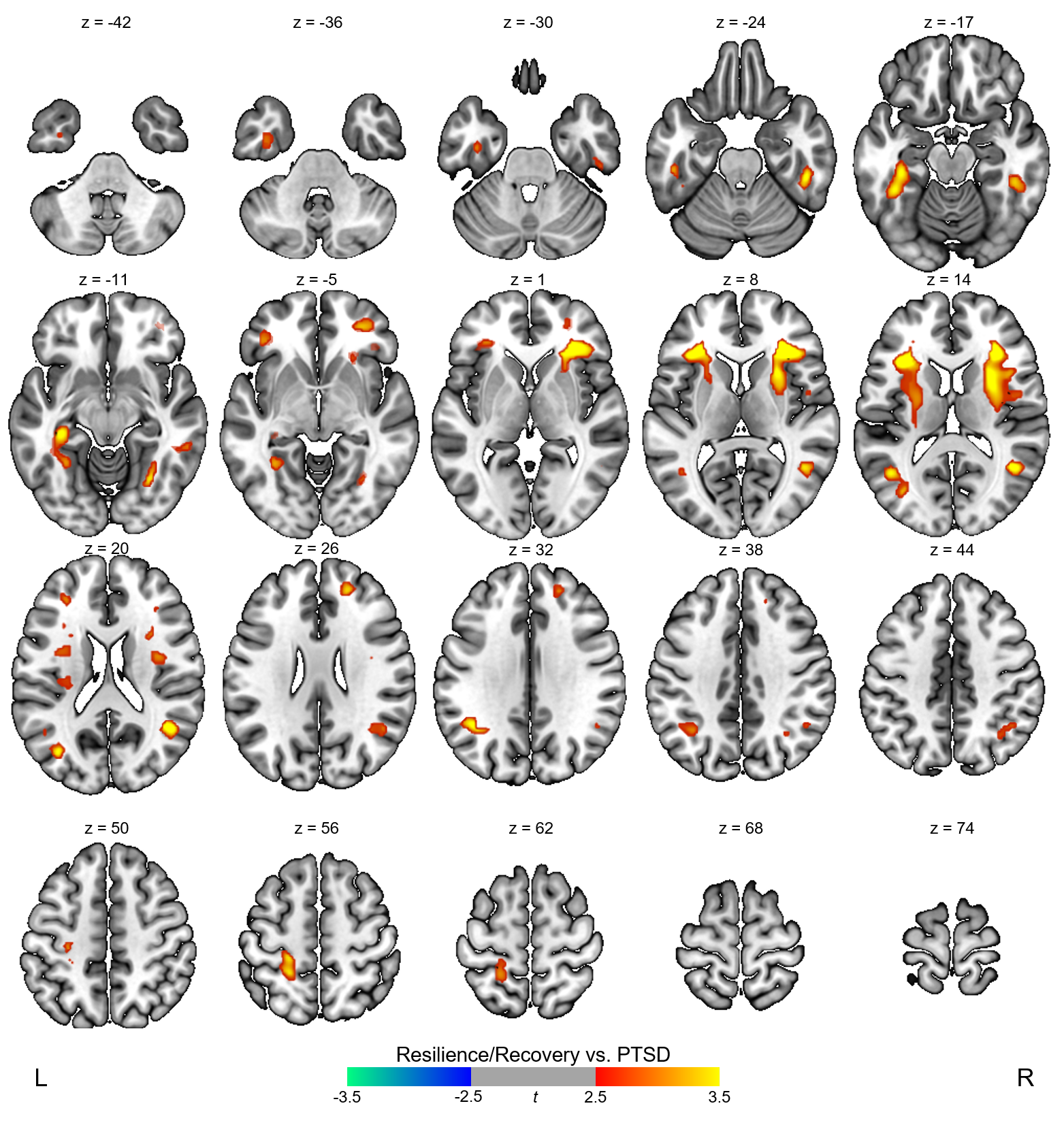


**Supplementary Figure 3.** Brain regions with higher (red-yellow) or lower (blue-green) regional cerebral metabolic rate of glucose in trauma-exposed individuals without current PTSD (Resilience/Recovery group) in comparison with those with current PTSD (PTSD group). An analysis of covariance was conducted with age and sex as covariates. The primary threshold was *p* < 0.005 and extent threshold was set at 100 or more contiguous voxels. The color bar represents voxel-level *t* values. The numbers above the brain slices indicate z coordinates in the Montreal Neurological Institute space. Detailed cluster information is listed in the Supplementary Table 3.

L, left; PTSD, posttraumatic stress disorder; R, right.


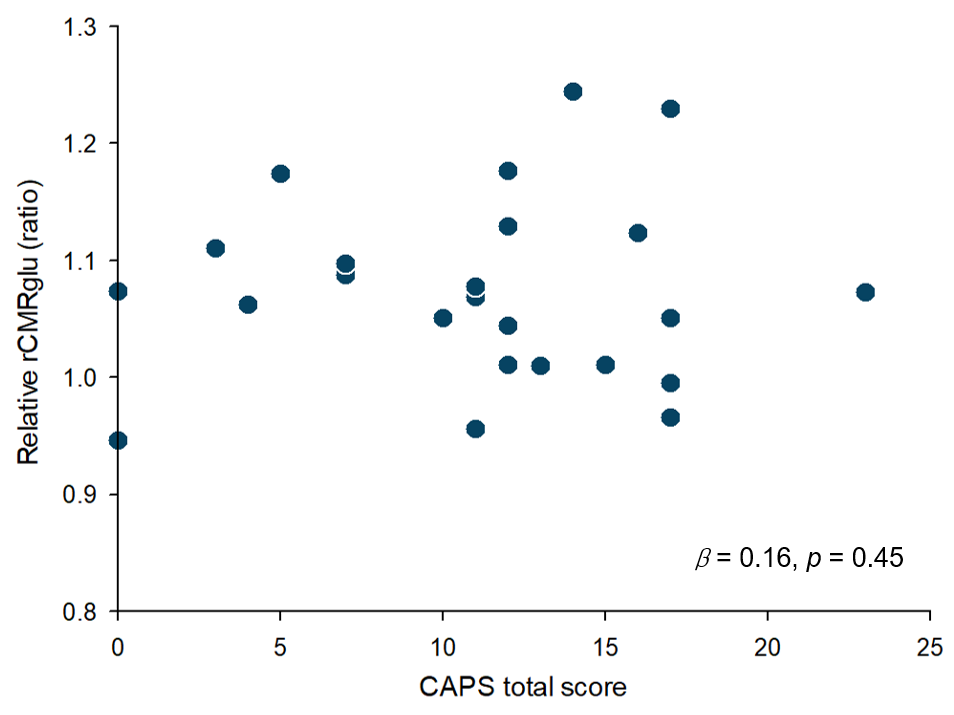


**Supplementary Figure 4.** Scatter plot to depict the association between the CAPS total scores and the relative rCMRglu of the right anterior insula cluster in the Resilience/Recovery group.

CAPS, Clinician-Administered PTSD Scale; PTSD, posttraumatic stress disorder; rCMRglu, regional cerebral metabolic rate of glucose.


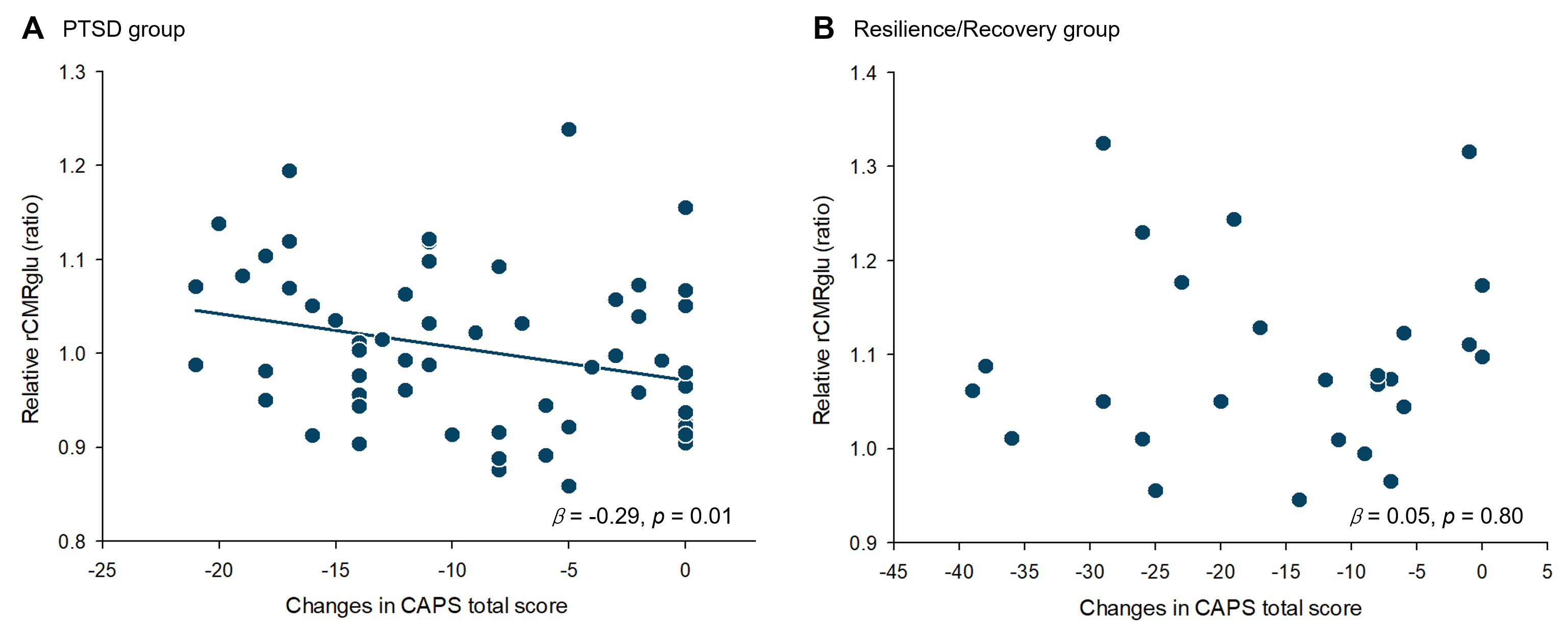


**Supplementary Figure 5.** Scatter plots to depict the association between changes in the CAPS total scores (current scores - lifetime scores) and the relative rCMRglu of the right anterior insula cluster in (A) the PTSD and (B) the Resilience/Recovery groups. The solid line indicates a regression line.

CAPS, Clinician-Administered PTSD Scale; PTSD, posttraumatic stress disorder; rCMRglu, regional cerebral metabolic rate of glucose.


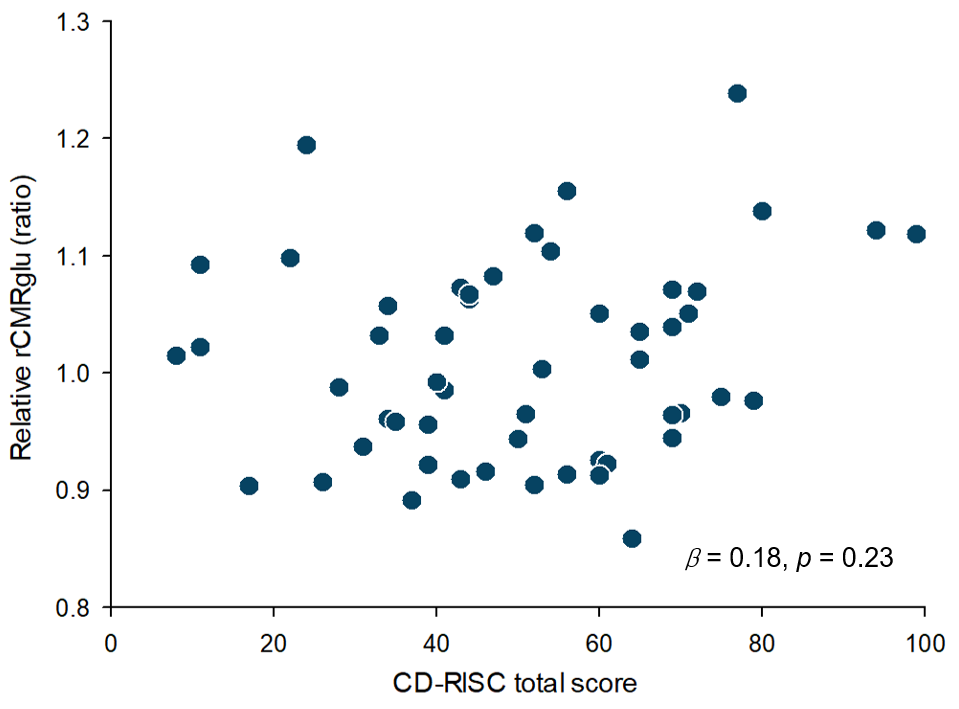


**Supplementary Figure 6.** Scatter plot to depict the association between the CD-RISC total scores and the relative rCMRglu of the right anterior insula cluster in the PTSD group.

CD-RISC, Connor‐Davidson Resilience Scale; PTSD, posttraumatic stress disorder; rCMRglu, regional cerebral metabolic rate of glucose.
